# In Vivo Transdermal Multi-Ion Monitoring with a Potentiometric Microneedle-Based Sensor Patch

**DOI:** 10.1021/acssensors.2c01907

**Published:** 2022-12-07

**Authors:** Águeda Molinero-Fernández, Ana Casanova, Qianyu Wang, María Cuartero, Gastón A. Crespo

**Affiliations:** † UCAM-SENS, Universidad Católica San Antonio de Murcia, UCAM HiTech, Avda. Andres Hernandez Ros 1, 30107Murcia, Spain; ‡ Department of Chemistry, School of Engineering Sciences in Chemistry, Biotechnology and Health, KTH Royal Institute of Technology, Teknikringen 30, SE-100 44Stockholm, Sweden

**Keywords:** multi-ion detection, microneedle sensor, wearable epidermal patch, multiplex intradermal analysis, in vivo measurements

## Abstract

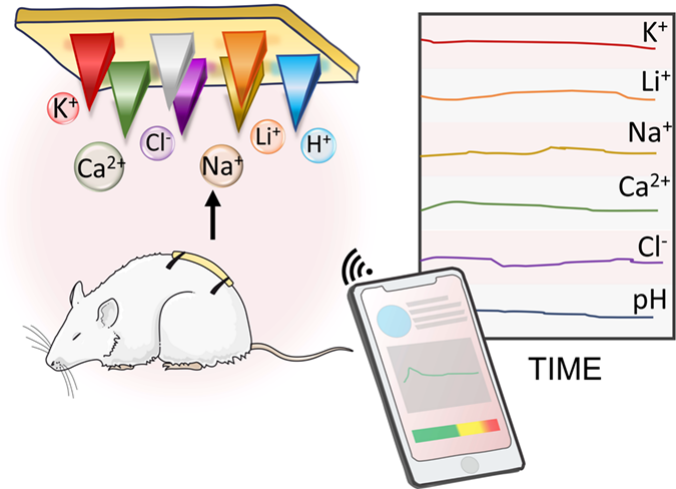

Microneedle sensor technology offers exciting opportunities for decentralized clinical analyses. A novel issue puts forward herein is to demonstrate the uniqueness of membrane-based microneedles to accomplish real-time, on-body monitoring of multiple ions simultaneously. The use of multi-ion detection is clinically relevant since it is expected to provide a more complete and reliable assessment of the clinical status of a subject concerning electrolyte disorders and others. We present a microneedle system for transdermal multiplexed tracing of pH, Na^+^, K^+^, Ca^2+^, Li^+^, and Cl^–^. The device consists of an array of seven solid microneedles externally modified to provide six indicator electrodes, each selective for a different ion, and a common reference electrode, all integrated into a wearable patch read in a potentiometric mode. We show in vitro measurements at the expected clinical levels, resulting in a fast response time, excellent reversibility and repeatability, and adequate selectivity. Close-to-Nernstian sensitivity, sufficient stability and resiliency to skin penetration guarantee the sensor’s success in transdermal measurements, which we demonstrate through ex vivo (with pieces of rat skin) and in vivo (on-body measurements in rats) tests. Accuracy is evaluated by comparison with gold standard techniques to characterize collected dermal fluid, blood, and serum. In the past, interstitial fluid (ISF) analysis has been challenging due to difficult sample collection and analysis. For ions, this has resulted in extrapolations from blood concentrations (invasive tests) rather than pure measurements in ISF. The developed microneedle patch is a relevant analytical tool to address this information gap.

Wearable chemical sensors may enable the digital transformation of clinical diagnostics. Invasive strategies focused on discrete blood tests will gradually be replaced by a new generation of analytical tools targeting peripheral biofluids. Features such as personalized, decentralized, and remote physiological/pathological conditions for the monitoring will be at the forefront of the “musts” to be addressed. In this context, interstitial fluid (ISF) analysis has been proposed as a promising strategy due to the similar compositions of ISF and blood.^1^ Microneedle-based (MN) sensors comprising needles with micrometric dimensions transformed to provide sensing capabilities are expected to measure ISF biomarkers in a minimally invasive and painless manner, anywhere, and without medical personnel intervention.^2^

Significant advances have been made in MN sensor development and application.^2^ It appears increasingly plausible to target the levels of a set of biomarkers, including electrolytes, with MN arrays toward a more complete health status monitoring, such as for electrolytes. For example, blood K^+^ and Na^+^ level changes are related to Li^+^ intoxication in bipolar disorder therapy.^3^ The Na^+^-to-K^+^ ratio has shown superior performance and reliability than either Na^+^ or K^+^ alone in evaluating hypertension.^4^ In addition, electrolyte balance is involved in basic life functions, such as body hydration, the generation of potentials and their propagation in muscles and nerves, and cell membrane transport.^5^ Several circumstances can lead to a deficit or excess of electrolytes, including loss of fluids, some medications, and underlying diseases, which may develop into dangerous health conditions such as heart rhythm disorders, convulsions, abdominal cramping, and even death.^6,7^

The electrolyte balance monitoring is recurrently accomplished in hospitals, from intensive care units to ambulatory rooms, using either point-of-care blood gas analyzers (direct blood measurements with potentiometric ion-selective electrodes [ISEs]) or auto-analyzers in central laboratories (diluted serum/plasma characterized with ISEs).^8,9^ Ion potentiometric transduction, but at the MN level to provide maximum decentralization, simplicity, and speed, seems to be essential to obtain health-related information. Therefore, several approaches have been proposed to transform MNs into potentiometric ISEs, especially for pH analysis. Inorganic material coatings (e.g., zinc oxide, iridium oxide, and molybdenum disulfide-polyaniline) have been applied to MNs for pH sensing and used in mouse bladders, rat hearts, and rodent brains.^10−12^ However, these approaches have not been transdermally proven, and there is a dearth of information on resiliency and potential toxicity effects.

Potentiometric MNs have been also conceived with plasticized polymeric ion-selective membranes (ISMs).^13−15^ This approach provides an attractive alternative due to the high biocompatibility of the materials used, simple fabrication, resiliency, low cost, and reliability. Therefore, ex vivo transdermal K^+^ was demonstrated in chicken skin^14^ and pH monitoring in euthanized rats.^14,15^ Membrane ISEs have also been integrated into microfluidic systems coupled to hollow MNs for K^+^ measurements in ISF. The biofluid was extracted by the MNs and directed toward a miniaturized porous carbon electrode covered with a K^+^-selective membrane (i.e., off-skin). Despite this system’s acceptable analytical performance, only in vitro assessment was performed.^16^ A dual MN system with potentiometric readout combining K^+^ and Na^+^ sensors inserted inside the same hollow MN was also reported. However, this concept is risky due to the proximity between the electrodes (i.e., the membranes are touching between them), potentially producing erroneous results.^17^ Despite notable advances in potentiometric MN sensor technology, the development and validation of a system for the simultaneous, in vivo monitoring of multiple ions has not yet been fully realized.

Here, we report the first MN sensor array for transdermal in vivo multi-ion assessment in ISF via a skin-worn patch. We present the characterization and application of the multi-ion MN (MIMN) patch for detecting pH, K^+^, Na^+^, Li^+^, and Cl^–^. First, analytical figures of merits are established (in vitro phase). After that, ex vivo assays were performed to demonstrate the suitability of the MIMN to perform skin penetration and reliable analytical quantification. Finally, in vivo (on-body) studies in euthanized rats were performed with additional analysis of collected blood/serum. The path followed to investigate the features of the MIMN patch (i.e., in vitro, ex vivo, and in vivo measurements) together with additional results based on gold standard techniques ensures the appropriateness of the data validation. Nevertheless, we strived for pure ISF measurements rather than inconvenient blood tests, overcoming current drawbacks that impede such a transition, including complex ISF collection methods, low sample volumes, and high risk of sample alteration during collection (pretreatments and analyses).

## Experimental Section

### Wearable MIMN Patch

**Figure 1:**
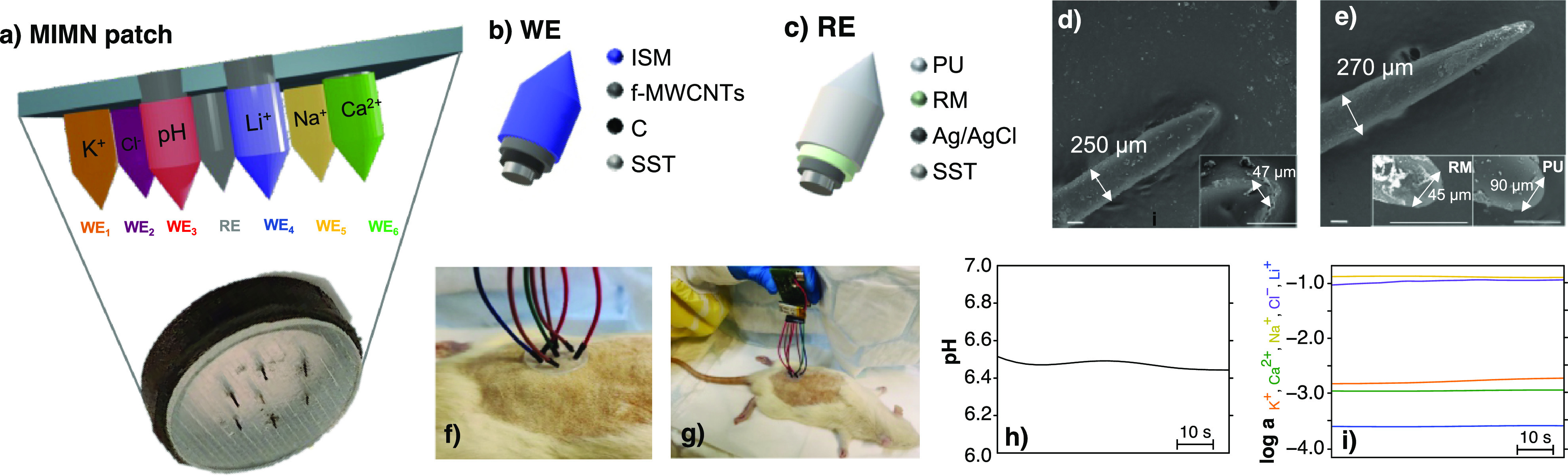
(a) MIMN patch. (b) Schematic of the WE layers. (c) Schematic of the RE layers. (d) SEM image of the WE for pH. Inset: magnification of the tip part. Scale bar: 100 μm. (e) SEM image of the RE. Inset: magnification of the tip part before (left) and after (right) the addition of the PU layer. Scale bars: 100 μm. (f) Image of the MINM patch inserted in a euthanized rat. (g) Image of on-body measurements in a euthanized rat with the MINM patch connected to the potentiometric board. Illustration of dynamic in vivo measurements obtained for (h) pH and (i) K^+^, Na^+^, Ca^2+^, Li^+^, and Cl^–^. Key: ISM, ion-selective membrane; PU, polyurethane; RM, reference membrane; SST, stainless steel.

Multi-ion measurements were achieved using a patch made of a silicone rubber substrate (Ecoflex 00-50 platinum cure; USA) with seven stainless steel MNs (1500 μm in length and 150 μm in diameter; Dermorroller) that were externally modified to create six working electrodes (WEs) and a common reference electrode (RE), Figure 1a. First, the MNs were dip-coated with the corresponding ink (i.e., carbon [C] for the WEs and Ag/AgCl for the RE) and cured in an oven (120 °C, 10 min). The MNs were inserted into the substrate and glued with Loctite Super Glue (Henkel Norden AB) to the upper MN part. This upper part was then used to make the electrical connections to the reader. After drying the glue for at least 4 h at room temperature, the remaining functionalization steps were accomplished in the bottom, sensing MN part.

For the WE, ten 2 μL layers of functionalized multiwalled carbon nanotubes (f-MWCNTs) were coated onto the carbon MN by drop-casting, as previously optimized.^14,15^ Drying steps of 4 min were performed between layers. Then, three 1 μL layers of the corresponding ISM cocktail were drop-casted. Drying steps of 20 min were performed after the first and second layers and 4 h after the final layer. Finally, the MNs were conditioned overnight in 10^–2^ M solutions of the corresponding analyte. For the RE, three 3 μL layers of the reference membrane cocktail, comprising a mixture of polyvinyl butyral (PVB) and NaCl in methanol, were placed onto the Ag/AgCl MNs. Drying steps of 20 min after the first and second layers and 4 h after the final layer were performed. After overnight conditioning in a 3 M KCl solution, the RE was dried for 1 h, and a layer of 2 μL polyurethane (PU) was drop-casted.

### In Vivo Measurements with the MIMN Patch in Rats

In vivo experiments with rats were conducted at the Karolinska University Hospital (Stockholm, Sweden) and assisted by the Operation Manager and personnel at the Karolinska Experimental Research and Imaging Centre (KERIC). Bio-breeding diabetes-prone rats used for other research purposes than those reported here (i.e., they were not euthanized explicitly for our investigations) were donated by KERIC. Prior to in vivo and ex vivo measurements, 5-point calibration curves covering the expected concentration range for each ion analyte were created. Then, each rat was euthanized in a CO_2_ chamber, and the MINM patch was manually inserted into the rat’s shaved back skin. After the on-body measurements, cardiac puncture blood collection was performed following established methods. Then, the rat’s back was opened by skin incision with a scalpel, and the subcutaneous tissue pH was measured with a micro-pH electrode (LL Biotrode; Metrohm, Nordic Sweden). Additionally, ISF was collected as reported elsewhere.^15^

## Results and Discussion

### MIMN Patch Definition

The MIMN patch consisted of six WEs, one for each analyte (WE_1_: K^+^; WE_2_: Cl^–^; WE_3_: pH; WE_4_: Li^+^; WE_5_: Na^+^; and WE_6_: Ca^2+^) and a common RE. Figure 1a shows a schematic of the MIMN patch and the color code used throughout the article (K^+^: orange; Cl^–^: purple; pH: red; Li^+^: blue; Na^+^: yellow; and Ca^2+^: green). A photo of the MINM patch supported on a rubber base for better handling is also provided. All WEs were all-solid-state ISEs based on a three-layer structure: (i) film made of carbon ink to improve conductivity and adherence of the other layers to the MN; (ii) f-MWCNTs as the ion-to-electron transducer; and (iii) the ISM (Figure 1b). A SEM image of the cross-section of one of the WE showed the assembly of the three layers (Figure S1). The carbon and f-MWCNT layers (with a thickness of ≈20–30 μm) deposited on the SST MN were completely covered with the polymeric ISM (<25 μm thickness). All the layers showed a relatively uniform thickness along the MN diameter. The membrane cocktail composition differed for each ion (Table S1); different selective receptors were used depending on the targeted ion. The RE was also an all-solid-state type and consisted of an Ag/AgCl ink layer covered by a PVB reference membrane (Figure 1c).^15^ An extra PU layer was finally added to the RE to improve the potential stability and resiliency to skin insertions.^14,15^

MN dimensions and geometry were characterized by scanning electron microscopy (SEM) as these are critical aspects to reach the ISF transdermally, resist skin penetration, and reduce the inflicted pain to the future user. Representative images of an MN-based WE and the MN-based RE are shown in Figure 1d,e, respectively. All the WEs had a length that was appropriate to reach the ISF (500 μm) while minimizing the pain to the user (base diameter < 300 μm, tip diameter < 50 μm, and tip angle < 45°). Similarly, the RE had a length of 500 μm, base diameter of <300 μm, tip diameter of ∼50 μm, and tip angle of 45°. Adding the PU layer to the RE increased its thickness by ∼20 μm without any significant tip angle alteration (inset in Figure 1e). According to previous studies, the modified MNs developed here fulfill the characteristics required for proper insertion into the skin while ensuring user comfort.^18^

The MIMN patch was conveniently designed to be worn on the skin and provide intradermal multi-ion measurements, as shown for rats in Figure 1f. It included cable-based connections from each MN to a potentiometer (e.g., a portable and miniaturized electronic board previously developed in our group;^15^Figure 1g). To provide on-body measurements, the MNs were calibrated, and the obtained graphs were used to convert the recorded potential (electromotive force [EMF]) into pH or the corresponding ion activity. Figure 1h,i show examples of dynamic in vivo measurements of pH and K^+^, Ca^2+^, Na^+^, Li^+^, and Cl^–^ activities in rats.

### In Vitro Analytical Characterization of the MIMN Patch

**Figure 2:**
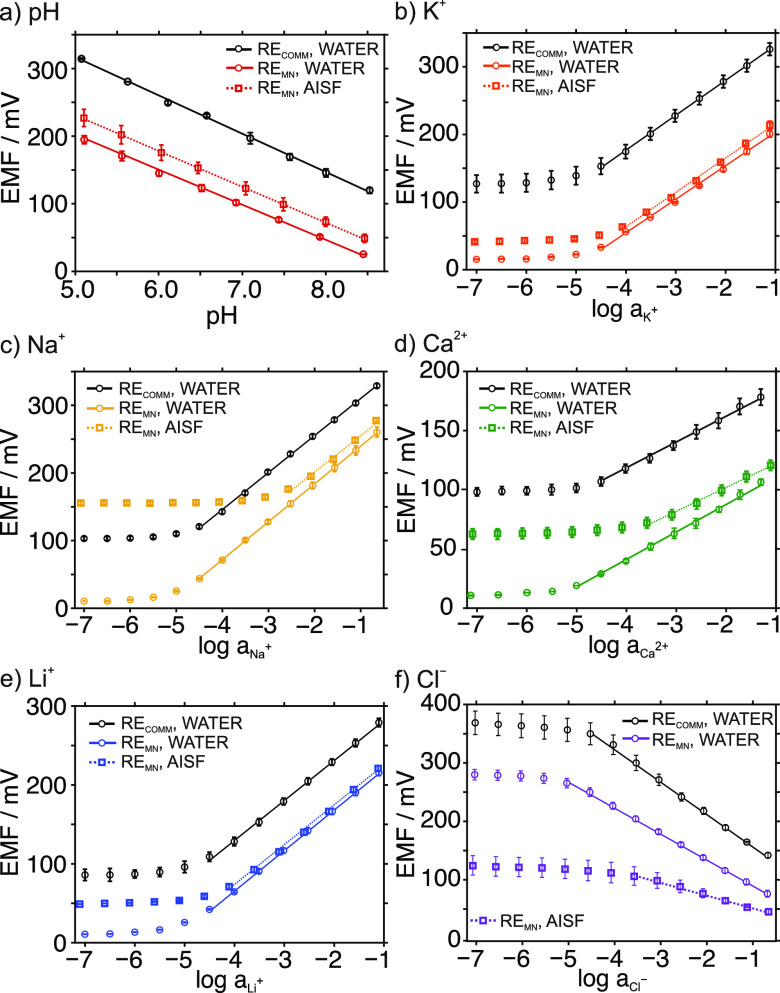
Calibration graphs obtained with the MN-based WEs (a) pH, (b) K^+^, (c) Na^+^, (d) Ca^2+^, (e) Li^+^, and (f) Cl^–^ against both the commercial Ag/AgCl RE (black) and the developed MN-based RE (color) in ultrapure water and artificial ISF backgrounds. Each point is calculated as the average of three measurements performed with the same WE, and the error bars represent the standard deviation for each average.

First, each MN-based WE was characterized at the in vitro level by regular measurements in a beaker (ultrapure water background) against either a commercial Ag/AgCl RE (RE_comm_) or the MN-based RE (RE_MN_) following the IUPAC recommendations (see the Supporting Information). Figure 2 shows the average calibration graphs obtained from three consecutive experiments for each ion analyte. As an example, one of the dynamic potentiometric responses observed at increasing concentrations of each ion is provided in Figure S2. The results using the RE_comm_ revealed close-to-Nernstian slopes without significant between-calibration difference, suitable limits of detection (LODs), and linear ranges of response (LRRs) that included the expected levels of the tested ions in the ISF in both humans and rats (Table S2). Fast response times were shown within the corresponding LRR by all the MN-based WEs (*t*_95_ < 5 s). The most relevant analytical parameters of the MIMN sensor are summarized in Table S3. In addition to fulfill the requirements for transdermal ion measurements in a real context, the MIMN presented comparable sensitivities, LODs, and LRRs to other MNs already published in the literature (Table S4). Although wider LRRs were reported by certain pH-MN sensors utilizing different materials as the sensing element, the MIMN covers the expected pH range for a real application.

Replacing the RE_comm_ with the RE_MN_ mainly translated into a variation in the intercept of the calibration graph (offset of ∼100 mV), decreasing for cations and increasing for Cl^–^, and the relatively good maintenance of the other analytical parameters (Table S3). Previous characterizations of analogous RE_MN_ showed acceptable response stability (over 16 h), absence of response to Cl^–^ salts and other compounds, no pH influence, and no effect of light/dark conditions.^14^ Altogether, our results showed that using the RE_MN_ did not influence the performance of the WE MN sensors. Therefore, the RE_MN_ was used for further measurements.

**Figure 3:**
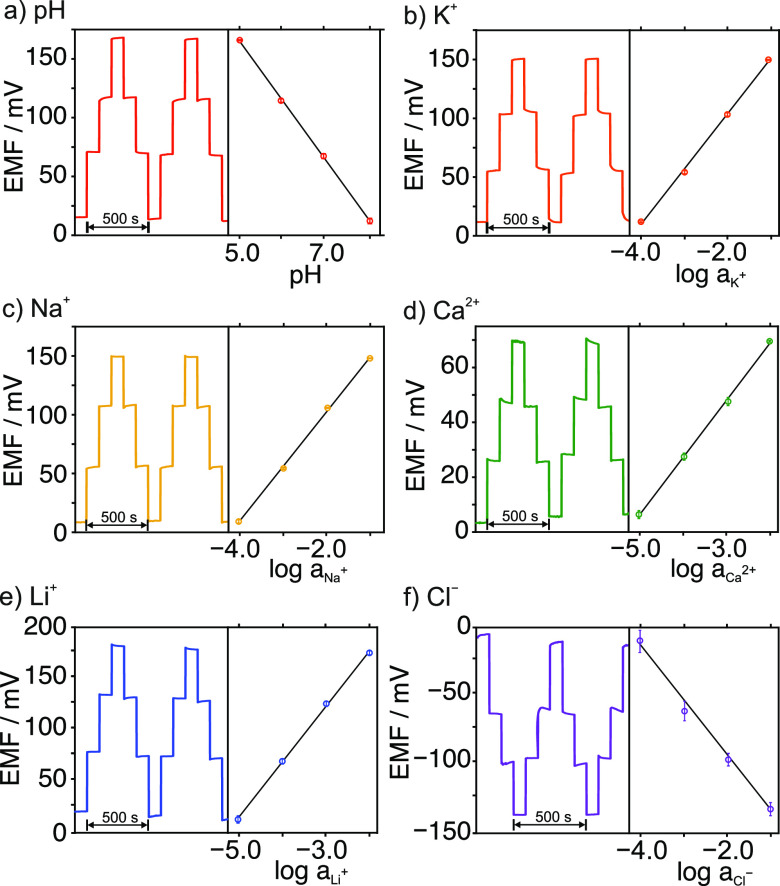
Reversibility study of the MIMN patch. Dynamic response and corresponding averaged calibration graphs for (a) pH, (b) K^+^, (c) Na^+^, (d) Ca^2+^, (e) Li^+^, and (f) Cl^–^.

Next, the performance of the MIMN patch was characterized as a whole. Response repeatability, calculated from three consecutive calibration graphs created with the same electrode, showed percentage relative standard deviations (%RSDs) of <8% and <9% for the slope and intercept for all the ions, respectively (Figure 2 and Table S5). Interelectrode reproducibility was evaluated using calibrations for three analogous MIMN patches, showing %RSDs of <10% for the slope and ∼10% for the intercept for all the ions (Figure S3 and Table S5). The reversibility within the LRR was evaluated from the results of four successive calibration curves performed alternating increasing and decreasing concentrations of each ion. The dynamic potential responses and the averaged calibration graphs are presented in Figure 3. Reversible signals were observed for all ions, except for lower Cl^–^ concentrations. A %RSD >30% was found for the EMF recorded for 10^–4^ Cl^–^ activity, while the other Cl^–^ activities had %RSDs of 4, 3, and 0.1%. For the other ions, %RSDs were <2.4% (Table S6). Remarkably, the reversibility study was conducted under very severe activity/concentration change conditions, which are not expected to occur during in vivo tests, but rather served to investigate the benefits and limits of the developed MIMN patch to provide the best accuracy.

The MIMN patch’s selectivity was evaluated by the separate solution method, in which individual calibration graphs are created for each WE’s interferences and primary analyte.^19^ The interferences tested the other cations for which each of WE is not selective but included as analytes in the MIMN. Notably, other ISF components, such as Mg^2+^, glucose, and urea, are not expected to affect the potentiometric response of MN sensors.^15^ A comparison of the calculated logarithmic selectivity coefficients with those theoretically required to perform potentiometric measurements without interference (Table S7) showed that the MIMN patch is, in principle, suitable for measuring the primary ions in real ISF without any potential interference. Moreover, the values agreed with those reported for other MNs for pH and K^+^,^14,15^ and ISEs based on the same ionophores are used here.

To further confirm the adequacy of the MN-based WEs to perform measurements in ISF without any chemical interference, calibration graphs for each ion were assessed in artificial ISF (AISF) and compared with those operated in ultrapure water (Figure 2 and Table S8). No significant differences in slopes were observed except for Cl^–^, while slightly higher LODs and narrower LRRs were obtained. These results remain sufficient for measuring pH, K^+^, Na^+^, Ca^2+^, and Li^+^ in the physiological and therapeutic range (Table S2). For Cl^–^, the slope showed a marked decrease, which indicates a matrix effect in the response. The LOD was higher and the LRR was narrower in AISF than in ultrapure water, same as the other ions. Acceptably, the MN for Cl^–^ can still be used for its measurement in ISF at the expected levels but with reduced sensitivity.

The stability of the MN’s response was tested in AISF over 15, 30, and 120 min periods, with more striking drifts observed over longer evaluation time (Table S9). While concentration variations of <5% were observed over short periods, they increased up to 74% for Ca^2+^ over longer time. Notably, any drift issue will strongly affect Ca^2+^, because of lower sensitivity (according to the Nernst theory) and lower ISF concentration than the other ions in ISF, and Li^+^, because of lower concentration and poorer selectivity of the sensor. These results are acceptable for accurate outcomes in this paper’s ex vivo and in vivo tests, which had a maximum duration of 1 min per skin insertion. Nevertheless, a measurement protocol based on pertinent reconditioning and recalibration of the MNs is convenient to enhance results reliability in long-term measurements. Importantly, in daily clinical practice, plasma electrolytes are measured by serial analysis (e.g., every 1 or 2 h for patients with hypernatremia or every 6 h for hypocalcemia, but shorter frequencies have been reported) of collected and pretreated blood.^6^ The new MIMN patch could replace these recurrent measurements through a less invasive and faster approach and provide more strict monitoring of specific clinical treatments and procedures, such as packed cell transfusion.^20^

### Ex Vivo Analytical Characterization of the MIMN Patch

**Figure 4:**
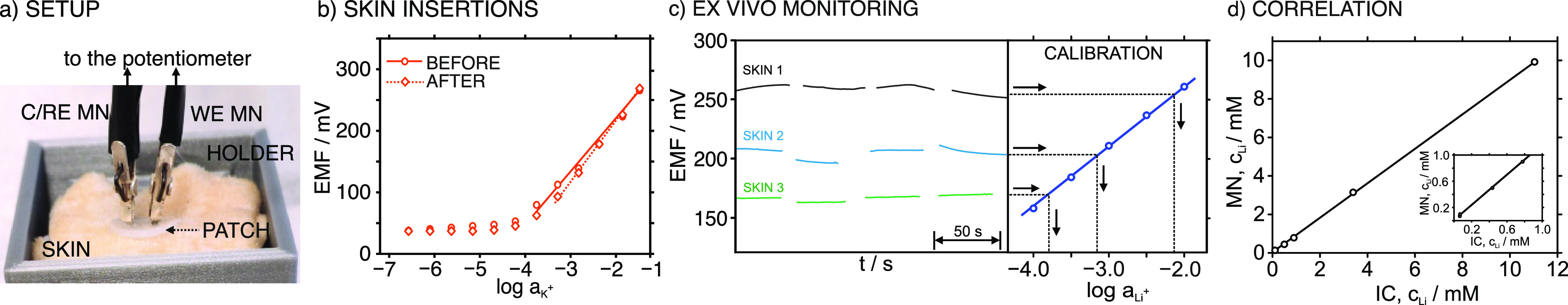
Ex vivo experiments. (a) Image of the experimental setup. (b) Calibration graphs for K^+^ before and after skin insertion. (c) Dynamic potentials observed for three rat skins conditioned with different Li^+^ concentrations. Four insertions are shown for each skin. Li^+^ concentration in the dermis fluid in each skin was calculated through extrapolation with a calibration graph. (d) Correlation between Li^+^ measurements provided by the MIMN and IC.

Before in vivo transdermal measurements, the resiliency of the MIMN patch to skin penetration was evaluated to confirm the absence of any alteration in the electrodes’ responses that might cause inaccurate results. Calibration graphs for each ion were created before and after three consecutive insertions in pieces of rat skin using a hand-made device (Figure 4a). The results observed for the K^+^ calibration before and after three skin insertions are presented in Figure 4b as an example. Table S10 lists the variations observed for the calibration parameters. No significant changes were observed in the slope or the intercept (%RSDs <6 and 10%, respectively). Indeed, these variations are within the levels obtained in the in vitro repeatability. Therefore, it is reasonable to not attribute them to the influence of skin insertion but to the inherent behavior of the MNs. Any physical alteration of the MNs or attachment of biological elements/tissue was refuted by comparing optical images of the same MN before and after skin insertion (Figure S4). No sign of deterioration or detachment of the sensing element was detected, which is consistent with the absence of associated variations in electrode calibration parameters after several insertions, indicating the absence of biofouling effects and demonstrating the maintenance of the MNs’ integrity.

Ex vivo tests were also used to validate transdermal measurements with the MIMN patch. Pieces of rat skin were conditioned overnight (at 4 °C) by immersion in solutions containing different concentrations of the target ions within the physiological range of interest. The final intradermal pH and the concentrations of K^+^, Na^+^, Ca^2+^, Li^+^, and Cl^–^ reached in the dermal fluid after the conditioning process were obtained with separate MN patches (containing a pair of WE and RE to measure the corresponding ion) and reference techniques analyzing the collected intradermal fluid with a micro-pH electrode and ion chromatography (IC). The potentiometric signals from several skin insertions were recorded with the same MN patch in the same skin piece, guaranteeing that the responses of at least three insertions were valid to provide averaged results for the corresponding pH or ion concentration. The dynamic readouts observed with the different MN patches were similar to those shown for Li^+^ in Figure 4c. The MN displayed different potential levels depending on the intradermal pH and ion concentration. Notably, the dynamic signals for each insertion were observed for ∼50 s. After recording the potentials, the intradermal concentration was calculated by extrapolating the averaged potentials into a previous in vitro calibration graph. For example, the entire procedure is shown for Li^+^ in Figure 4c.

As previously reported, the final intradermal ion concentration induced by a conditioning-based process depends on the concentration used in the solution, the conditioning time and the diffusion characteristics of the ion, history of the skin sample, thickness, fat level, and original body part.^14^ What was crucial was the achievement of different intradermal pH and concentrations for all the tested ions and quantifying the levels with both the MNs and the corresponding analytical reference method (Table S11). For the latter, the intradermal fluid was collected for 10 min by means of a custom-made system based on a hollow MN-hub previously reported by our group for ISF collection^15^ and analyzing the sample by a micro-pH electrode and IC. In all cases, the percentage difference between the inputs from the MN patch and the reference method was <20%, confirming the acceptable accuracy of the measurements provided by the MNs and encouraging its further use for in vivo measurements in rats.

The measurement of endogenous Li^+^ levels (0.14–8.6 μM and always <15.8 μM in human serum)^21^ was not expected in our posterior in vivo measurements in rats because the MIMN patch responds with an LRR within Li^+^ levels expected for therapeutic/prophylactic treatments (3 orders of magnitude higher than endogenous Li^+^). None of the rats analyzed here were under specific Li^+^ treatment. Therefore, the MN detecting Li^+^ in the MIMN was not properly validated in vivo. Consequently, the determination of Li^+^ was studied in the ex vivo phase using more samples than the other ions. Figure 4d shows the correlation between the Li^+^ concentrations provided by the MN patch and IC (*n* = 6). We found excellent agreement (*y*-intercept = 0.002, slope = 1.1) and a statistically significant positive correlation (Pearson’s coefficient = 0.99; *p* < 0.01).

### In Vivo On-Body Measurements with the MIMN Patch in Euthanized Rats

The ability of the MIMN patch to provide on-body transdermal measurements of multi-ion concentrations was assessed using six euthanized rats. The maximum time a rat wore the MIMN patch during each insertion was ∼2 min, avoiding any potential cytotoxicity risk caused by compound leaching from the membrane or response deterioration (drift). Remarkably, potential cytotoxicity was reported by previous studies using analogous MNs to those developed here when exposed to human dermal fibroblasts for >24 h.^13^ Their viability test results showed that valinomycin (K^+^ ionophore) and nonactin (NH_4_^+^ ionophore) were toxic to fibroblasts after they leached from the membrane into the culture media due to inhibiting cell proliferation rather than inducing apoptosis.

The rats involved in this study were of different ages and sexes (Table S12) to broadly cover histological variations in the skin and investigate the versatility of the developed MIMN patch. The MIMN patch was manually inserted into the back of each rat, which was shaved to facilitate appropriate penetration after euthanasia (Figure 1f,g). Electrical connections were made, and the MNs’ responses were acquired until a steady-state potential was present for at least ∼30 s. Dynamic EMF readings were transformed into concentrations according to the previous calibrations. Altogether, since these were the first in vivo multi-ion measurements in rats with the MIMN patch, we encountered some practical issues that must be resolved before its appropriate future use. For example, the insertion of the MNs might be facilitated by using an appropriate applicator rather than being manually accomplished (increasing research has recently been performed on applicator designs for MN arrays^22^) to ensure proper skin penetration and avoid excessive force that can damage the patch. In addition, the nature of the patch substrate should be investigated in the direction to be fixed in a platform compatible with the applicator for improved portability. Better definition of the region of the rat back/dorsal for the patch implantation; and miniaturized and lighter connections from the MNs to the electronic board will be also necessary. Importantly, our experiments provide in-depth ex vivo validation and accuracy confirmation and demonstrate the potential of the MIMN patch for chemical digitalization of physiologically relevant information in the subcutaneous ISF through in vivo (on-body) tests in rats.

In vivo measurements in rats were accompanied by ISF and blood sample collection to validate the on-body results. Adequately developed models based on these measurements are likely to advance the technological readiness of the MIMN patch toward human-based trials as preliminary steps to establishing clinical products. Unfortunately, validation is a complex task, and most previous articles on MN (bio)sensors lack these results and their related conclusions, as previously claimed.^23^ The mentioned limitations must be overcome investigating the next-generation MN sensor devices.

**Figure 5:**
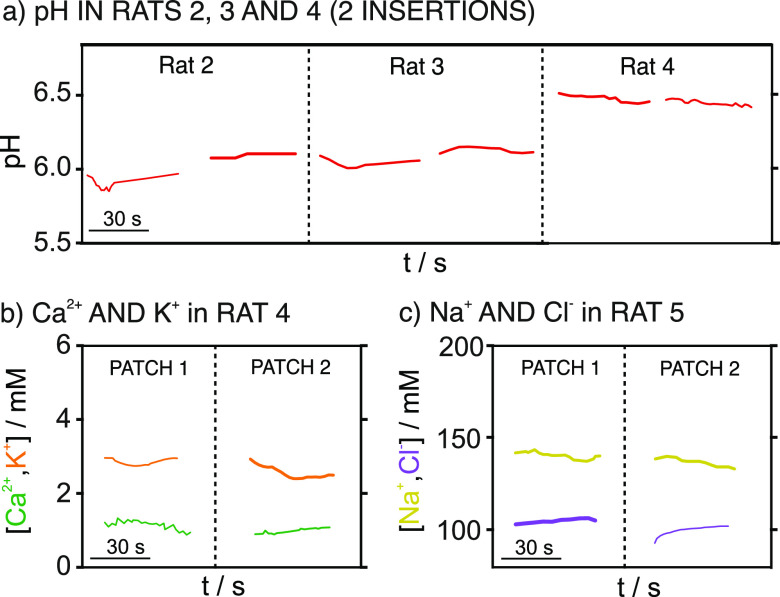
(a) Dynamic pH profiles for rats 2, 3, and 4 with two consecutive insertions of the same MIMN patch. (b) Dynamic Ca^2+^ and K^+^ concentration profiles for rat 4 with two analogous MIMN patches. (c) Dynamic Na^+^ and Cl^–^ concentration profiles for rat 5 with two analogous MIMN patches. Different patches were used for each rat to avoid contamination.

Some examples of the dynamic concentration profiles observed with the MIMN patch in several rats are presented in Figure 5. Stable and reasonably reproducible results were obtained for subsequent nearby insertions in the rat’s back (e.g., Figure 5a for pH) and when using different analogous MIMN patches (e.g., Figure 5b for Ca^2+^ and K^+^ and Figure 5c for Na^+^ and Cl^–^). Nevertheless, it should be noted that noisier and less stable signals were observed with increasing insertions using the same MIMN patch. Indeed, when measurements deviated from the initial measurements, they were not used for the concentration calculation. Inappropriate skin insertions, noisy and unstable signals, irreproducibility, and random EMF recording outside the calibration graph were the primary reasons for discarding data.

Table 1 lists the pH and ion (Na^+^, K^+^, Ca^2+^, and Cl^–^) concentrations in subcutaneous ISF of rats obtained with the MIMN patch. In addition, blood and serum levels are provided, enabling the calculation of the following ratios (*r*):
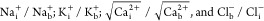
1where i refers to ISF and b refers to blood or serum. The values for these ratios are also reported in Table 1. Because a semipermeable membrane separates blood and ISF, the relationship between each ion concentration (pure activity) should correspond to the values predicted by the Gibbs–Donnan equilibrium.^24,25^ A theoretical Donnan ratio of 0.95 was established for activity proportionality of monovalent cations in ISF and plasma/serum in healthy rats and humans. For divalent cations, which are partially protein bound, the Donnan ratio was established as 0.90.^25^

**Table 1:** pH and Ion (Na^+^, K^+^, Ca^2+^, and Cl^–^) Concentrations in Subcutaneous ISF of Rats^a^

analyte	rat	concentrations (mM^b^)		
		ISF, MIMN	blood	serum
pH	1	6.0 ± 0.2	6.4	
	2	5.8 ± 0.2	7.2	
	3	6.1 ± 0.1 (5.9)	6.8	
	4	6.4 ± 0.7 (6.5)	7.0	
	5	6.7 ± 0.1 (6.0)	6.6	
	6	7.2 ± 0.4	6.5	
average ± SD		6.4 ± 0.5; *n* = 6 {*p* = 0.44}	6.8 ± 0.3; *n* = 6 {*p* = 0.17}	
Na^+^	1			
	2	159.3 ± 28.9 [1.14; −17%]		139.6
	3			121.8
	4	169.2 ± 9.0 [1.03; −6%]	164.0	
	5	149.0 ± 7.8 [1.1; −14%]	134.0	130.4
	6	110.0 ± 6.9^c^	136.0	133.7
average ± SD		146.9 ± 25.9; *n* = 4	144.7 ± 16.8; *n* = 3 {*p* = 0.90}	131.4 ± 7.4; *n* = 4 {*p* = 0.37}
K^+^	1	6.8 ± 0.9		
	2	2.0 ± 0.5		14.0^d^
	3			13.5^d^
	4	3.4 ± 0.1	13.1^d^	10.6^d^
	5	4.4 ± 0.3	13.6^d^	14.9^d^
	6	4.8 ± 0.6	17.1^d^	15.1^d^
average ± SD		4.3 ± 1.8; *n* = 5	14.6 ± 2.2; *n* = 3	13.5 ± 2.1; *n* = 4
Ca^2+^	1			
	2			
	3			
	4	1.3 ± 0.3 [0.93; +7%]	1.5	
	5	7.6 ± 0.7^d^	1.7	
	6	0.8 ± 0.1 [0.71; +19%]	1.6	
average ± SD		1.1 ± 0.4; *n* = 2	1.6 ± 0.1; *n* = 3 {*p* = 0.11}	
Cl^–^	1			
	2			103.9
	3	102.6 ± 13.1 [1.12; −14%]		114.6
	4	180.2 ± 10.2^d^	96.0	93.8
	5	106.1 ± 8.7 [0.98, −0.3%]	102.0	103.9
	6	136.4 ± 1.2^d^	101.0	105.5
average ± SD		104.4 ± 2.5; *n* = 2	99.7 ± 3.2; *n* = 3 {*p* = 0.18}	104.3 ± 7.4; *n* = 5 {*p* = 0.99}

a Blood and serum levels are also shown. Values in brackets indicate the pH measured via the micro-pH electrode in the subcutaneous tissue or the concentration ratios and their percentage deviations from a Donnan ratio. Values in braces indicate the *p*-value obtained from a Student’s t-test, where a *p* < 0.05 was considered statistically significant, comparing ISF values against blood/serum.

b pH is expressed in pH units.

c Value is slightly lower than previously reported data.

d Value is slightly higher than previously reported data.

We hypothesized that the Donnan ratio from blood/serum might be a proxy of in vivo validation of on-body ion measurements in ISF since any deviation may be due to (i) the presence of proteins in the analyzed fluids, (ii) the use of concentration instead of the formal activity, and (iii) our use of postmortem data. This hypothesis emerged from the practical difficulty of performing ISF sampling and analysis in the studied rats. Some ISF volumes (<5 μL) were obtained only for two out of the six rats, the youngest ones, likely because smoother skin and higher hydration facilitated collection.^26^ Moreover, as we progressed in the rat experiments and identified the true complexity of the validation strategy, we implemented subcutaneous pH measurements via an incision in the rat’s back with a microelectrode,^15^ blood measurements with a clinical gas analyzer, and serum measurements with the IC (Table S12).

Inspecting first the results for pH (Table 1), except for rat 6, the values were lower than expected, given their physiological range (Table S2). Indeed, pH measurements in blood were also lower than expected, coinciding with some reports on blood acidification when CO_2_ euthanasia was employed.^27^ Interestingly, blood acidification has been studied as a potential marker for postmortem interval, and values as low as 5.1 have been observed.^28^ In addition, it has been reported that even with blood showing normal pH (7.40 ± 0.05), the pH of ISF can deviate from the physiological range in patients with certain diseases, such as diabetes.^29^ Therefore, blood and ISF pH may not correlate, and ISF pH appears to provide clinical information that is not accessible from blood. However, when comparing the average pH of all six rats, which is a common procedure in clinical studies, no significant difference was observed between ISF pH measured with the MIMN and subcutaneous measurements with the pH-microelectrode (*p* = 0.44) or blood (*p* = 0.17). On the other hand, it would be normal to find significant differences as explained above. While measurements with the pH-microelectrode were a good alternative for the validation of our MIMN results, this would be unsuitable for investigations based on living animal or human subjects due to the high level of invasiveness.

The Na^+^ concentration observed for rat 6 with the MIMN was slightly lower than previously reported values for rats and those observed in blood and serum. Rats 2, 4, and 5, for which we obtained a complete set of ISF and blood/serum data, the concentrations were relatively close in all the analyzed fluids. Furthermore, the calculated Donnan ratios were close to the theoretical value of 0.95 (deviations of <17%). Potential deviations may arise from individual correction factors for plasma proteins having not been performed. Altogether, blood/serum measurements showed the accuracy of Na^+^ detection with the developed MIMN. In addition, given a *n* = 3 for the MIMN (rats 2, 4, and 5) and blood (rats 4–6) and four for serum (rats 2, 3, 5, and 6), no significant difference was observed in Na^+^ ISF measured by the MIMN compared to both blood (*p* = 0.90) and serum (*p* = 0.37).

In the case of K^+^, while all the measurements with the MIMN in the ISF were within the expected physiological range, those in blood and serum were rather high (2.0–6.8 mM vs 10.6–17.1 mM). Interestingly, blood K^+^ levels higher than the physiological range were previously observed in rodents euthanized by CO_2_.^27^ However, this effect appears to not manifest in the ISF, at least within the time frame of our experiments. Experiments involving K^+^ measurement with the MIMN avoiding CO_2_ euthanasia or other conditions that may affect its comparison with blood K^+^ are advisable in future research, including validation. On the other hand, the MIMN patch could potentially be used to follow anesthesia-related issues in the rat.

The Ca^2+^ levels in ISF and blood were somewhat similar to the expected physiological level, except for rat 5, whose Ca^2+^ concentration was abnormally high. Therefore, considering only the data for rats 4 and 6, no significant difference was presented between ISF and blood levels (*p* = 0.11). Furthermore, deviations for the Donnan ratio were acceptable. Notably, serum measurements in IC were not possible because of the deproteinization sample process, which intrinsically decreased the Ca^2+^ concentration. Nevertheless, blood measurements indicate good accuracy of the results provided by the MIMN for Ca^2+^.

Finally, there was good agreement between ISF and blood/serum Cl^–^ measurements in rats 3 and 5, while Cl^–^ concentrations in ISF obtained for rats 4 and 6 were relatively high considering the expected physiological range and calculated values for blood/serum. When the averaged Cl^–^ concentration in ISF was compared with those in blood and serum, no significant differences were observed (*p* = 0.18 and *p* = 0.36, respectively). Acceptable deviations were found for the calculated Donnan ratios in rats 3 and 5. Altogether, these results suggest that blood/serum measurements are a good alternative for validating in vivo Cl^–^ measurements with the MIMN.

A fundamental aspect that we consider essential to discuss concerns the use of concentration instead of activity values both in the calibration of MN ion sensors and the provision of the final levels in the biofluid. On one hand, because potentiometric ISEs formally respond to activity rather than concentration, a series of calculations and assumptions to apply the Debye–Hückel theory are necessary to convert both the calibration graph and the concentration results to activity and vice versa. Therefore, this remains a source of error affecting the final outcomes.^30^ On the other hand, it would be advantageous to provide and investigate the results on ion levels directly in activity terms to compensate for any protein influence in the related fluid, enabling a better study of correlation and disease relations.

## Conclusions

A fully validated MIMN patch for determining clinically relevant electrolytes and pH in ISF was developed. After a deep in vitro and ex vivo characterization, the MINM patch demonstrated high applicability for on-body transdermal digitalization of pH and electrolyte levels in euthanized rats. While ion measurement is a routine task in the clinical domain, information from ISF analysis is lacking and relies on theoretical calculations from blood test results. The MN sensor technology reported here can overcome the absence of reliable analytical tools for ISF analysis, generating new clinical information in upcoming studies.

## Supporting Information Available

Experimental details; analytical performances; ex vivo, in vivo results; stability and resiliency; and microscopy images (PDF)
