# Supplementary material for: In Vivo Transdermal Multi-Ion Monitoring with a Potentiometric Microneedle-Based Sensor Patch
Source: ACS Sens. 2022 Dec 7;8(1):158–66. doi: 10.1021/acssensors.2c01907 (PMC9887649; doi:10.1021/acssensors.2c01907)
Supplement: Supplementary file 1 — se2c01907_si_001.pdf [file se2c01907_si_001.pdf]

Supporting Information for:

In Vivo Transdermal Multi Ion Monitoring with a Potentiometric Microneedle-based Sensor Patch

Águeda Molinero-Fernández[b], Ana Casanova[b], Qianyu Wang[b], María Cuartero[a,b*], Gastón A. Crespo[a,b*]

[a]*UCAM-SENS, Universidad Católica San Antonio de Murcia, UCAM HiTech, Avda. Andres Hernandez Ros 1, 30107, Murcia, Spain.*

[b]*Department of Chemistry, School of Engineering Sciences in Chemistry, Biotechnology and Health, KTH Royal Institute of Technology, Teknikringen 30, SE-100 44, Stockholm, Sweden.*

Corresponding author (*): mariacb@kth.se; gacp@kth.se

Table of Contents

Experimental Section 3
- Reagents, materials and instrumentation. 3
- Fabrication of the MIMN patch. 4
- Potentiometric measurements. 4
- Selectivity studies. 4
- The use of the MIMN patch in euthanized rats. 5
- ISF collection in pieces of rat skin and euthanized rats. 5
- IC measurements. 5

Tables 6
- Table S1. 6
- Table S2. 6
- Table S3. 6
- Table S4. 7
- Table S5. 7
- Table S6. 7
- Table S7. 8
- Table S8. 9
- Table S9. 9
- Table S10. 10
- Table S11. 10
- Table S12. 11

Figures 12
- Figure S1. 12
- Figure S2. 12
- Figure S3. 13
- Figure S4. 13

References 14

Experimental Section

Reagents, materials and instrumentation. Hydrogen ionophore I (selectophore grade), potassium ionophore I (valinomycin, selectophore grade), sodium ionophore X (selectophore grade), calcium ionophore IV (selectophore grade), lithium ionophore VI (selectophore grade), sodium tetrakis[3,5 bis(trifluoromethyl)phenyl]borate (NaTFPB, > 98%), potassium tetrakis (4-chlorophenyl) borate (KTClPB, > 98%), tridodecylmethylammonium chloride (TDMACl, > 98%), polyurethane (PU), bis(2-ethylhexyl)sebacate (DOS, > 97%), 2-nitrophenyl octyl ether (NPOE, > 99%) and tetrahydrofuran (THF, > 99.9%) were purchased from Sigma Aldrich. Analytical grade chloride salts of potassium, sodium, calcium, magnesium and lithium as well as sodium carbonate, sodium phosphate, sodium citrate, bovine serum albumin (BSA), urea and glucose were purchased also from Sigma-Aldrich. Carbon ink (C2030519P4) and silver/silver chloride (Ag/AgCl) 50/50 paste (C2131007D3) were obtained from Sunchemical. All solutions were prepared using 18.2 MΩ·cm doubly deionized water (Milli Q water systems, Merck Millipore).

Synthesis of lipophilic functionalized multiwalled-carbon nanotubes (f-MWCNTs) was performed as reported elsewhere. For this purpose, thionyl chloride ($SOCl_2$ ≥ 97% purity), octadecylamine (ODA, > 99%), dimethylformamide (DMF, > 99%) and MWCNTs (from Sigma Aldrich) were used.[1] Chloride ionophore (1,1',1''-(nitrilotris(ethane-2,1-diyl))tris(3-(3,5-bis(trifluoromethyl)phenyl)urea)) was synthesized according to the protocol described in the literature.[2]

The artificial interstitial fluid was prepared with 3.5 mM KCl, 1.5 mM $CaCl_2$, 0.7 mM $MgCl_2$, 140 mM NaCl, 26 mM $NaHCO_3$, 1.7 mM Na_2HPO_4, 6 mM glucose and 7 mM urea. When measuring the calibration of one ion, this was omitted from the recipe.

Silicon rubber (Ecoflex 00-50 platinum cure, USA) and stainless-steel microneedles (MN) (Dermaroller local supplier, Sweden) were employed for the fabrication of the MN patch.

For the ex vivo experiments, rat skins of euthanized specimens at KERIC were cut into squared pieces and stored in the freezer at –18 °C. Before being used, each piece of skin was defrosted at room temperature and gently cleaned with distilled water.

Electromotive force (EMF) was measured with a high input impedance (1015) EMF16 multichannel data acquisition device (Lawson laboratories, Inc.) against a double junction Ag/AgCl/sat. A hand-made potentiometric board with wireless data transmission was used for the rat-based experiments at Karolinska Institute Hospital.[3] A double-junction Ag/AgCl/sat. KCl/1 M LiOAc reference electrode (6.0726.100, Metrohm Nordic, Sweden) was also used.

Scanning Electron Microscopy (SEM) images were taken with a Hitachi TM-1000 Tabletop SEM (Hitachi High-Tech, Inc.). Optical microscopy images were taken using a Nikon Eclipse Ti2 inverted optical microscope coupled to a DS-Qi2 camera (Nikon Instrument, Inc.). An 850 Professional IC ion chromatography system coupling with IC conductivity detector (Metrohm Nordic, Sweden) was employed for ions measurements in interstitial fluid samples. Metrosep C6 150/4.0 (6.1051.420) column for cation analysis and Metrosep A Supp 5 105/4.0 (6.1006.520) column for anion analysis were employed.

Micro-pH meter (LL, biotrode, Metrohm, Nordic Sweden), and ultra-micro pH meter (Orion, Ultra-Micro Combination pH Electrode, Thermo Scientific) were used for subcutaneous and extracted ISF pH measurements, respectively.

ABL90 FLEX PLUS blood gas analyzer (Radiometer, Denmark) was employed for ions quantification in blood.

Fabrication of the MIMN patch. The MIMN patch consisted of six MN-WEs (one for each analyte) and one MN-RE inserted on a silicon rubber subtrate. The MNs used in these experiments have a full length of 1.5 mm and a diameter of 250 µm. The patch substrate was fabricated by mixing equal volumes of the solutions, labelled as "Part A" and "Part B", in the commercial pourable silicon rubber kit (Smooth-on, USA) and filling a 3D printed mold of 23 mm of diameter and 1 mm of depth with the resulting mixture. Then, the substrate was allowed to cure for 3 h, according to the manufacturer instructions. Prior to the insertion into the substrate, the stainless-steel MNs were coated with carbon or Ag/AgCl ink for the working electrodes and the reference electrode, respectively. Afterwards, the MNs were attached on the substrate with Loctite Super Glue (Henkel Norden AB), which was left to dry at room temperature for 4 h. Finally, the functionalization with the ISMs was performed for each MN.

Working electrodes: Regardless of the analyte, the stainless-steel solid MN was coated with the carbon ink and the created film was allowed to cure in the oven at 120 °C for 10 min. Once room temperature is reached, the C-MNs were fixed in the substrate and the f-MWCNTs film was added by drop-casting (10 layers of 2 μL) a dispersion of f-MWCNTs in THF (1 mg/mL), with 4-min drying at room temperature in between layers. Finally, the corresponding ISM (composition collected in **Table S1**) was implemented by drop casting (3 layers of 1 μL), allowing each layer to dry 20 min at room temperature before the addition of the following one. The final film was dried for 4 h before conditioning. The working electrodes were conditioned overnight in 10^{-2} M solution of the corresponding analyte. For the pH working electrode, it was conditioned in 10^{-3} M of HCl. Analytical parameters were calculated according to IUPAC recommendations for ISEs.[4, 5]

Reference electrode: An Ag/AgCl film was deposited on top of the stainless-steel MN by dip coating of the commercial Ag/AgCl ink and, successively, the film was cured in the oven at 120 °C for 10 min. After appropriate fixation in the substrate, the reference membrane cocktail was drop-casted (3 layers of 3 µL) on top of the Ag/AgCl film. Each layer was allowed to dry at room temperature for 20 min before drop casting the next one. Then, the last layer was dried for 4 h before an overnight conditioning in 3 M KCl. Finally, the MN-RE was dried at room temperature for 1 h and a volume of 2 µL of polyurethane was drop casted on top of the MN and left it to dry for 4 h. The aim of this last layer was avoiding the salt (KCl) leaching out and improving the potential stability of the MN-RE.[6]

Potentiometric measurements. Calibration experiments were carried out at room temperature (22 ± 1 °C) under constant stirring of 300 rpm (stirrer IKA COLOR SQUID S000, IKA, Germany). The MNs in the MIMN patch were connected to the potentiometer by a cable based on electrical clamps and BNC outputs. The activity coefficients to be used in the calibration graph were calculated using a two-parameter Debye Hückel approximation from the experimental concentrations.[7] Each logarithmic activity was plotted against the corresponding steady-state potential, and the curves were fitted to the Nernst equation.

Selectivity studies. Selectivity was evaluated using the separate solution method (SSM) according to Bakker et al. Individual calibration graphs were accomplished for the primary and the interfering cations, and the logarithmic selectivity were calculated by extrapolating the response to $a_I = a_J = 1$ using the portion of the calibration curve close to Nernstian response.

The use of the MIMN patch in euthanized rats. The potentials of the MNs in the MIMN patch were recorded with a hand-made potentiometric electronic board equipped with a Bluetooth low energy system able to take up to 900 samples per second.3 After the measurements, cardiac puncture blood collection was performed following established methods. After that, once rat backs were opened with a scalpel, pH was measured from the ISF in the exposed subcutaneous tissue by a micro-pH meter for pH measurements validation purposes. ISF and blood samples were additionally collected. Serum was obtained by placing the corresponding blood extraction tube in an upright position at room temperature to allow the blood to clot (15 – 30 min). After that, the clot was removed by centrifuging at 1100 rpm for 10 minutes. For IC measurements, deproteinization was performed by mixing the serum with ethanol (31.5% v/v) for 30 minutes. The supernatant was removed after centrifugation at 1100 rpm for 5 minutes.

ISF collection in pieces of rat skin and euthanized rats. ISF was collected by using a homemade device consisting in a plastic hub containing 4 hollow microneedles (0.24 x 0.11 x 10 mm, Micropoint Technologies Pte Ltd, Singapore). The hollow length exposed in the hub was 1.5 mm and it was coupled with a microfluidic PFTE tubing (Sigma-Aldrich) with 0.3 and 0.6 mm of inner and outer diameter, respectively. A time of 10 min extraction was assisted by means of a tubing (Tygon LMT-55, ISMATEC, Cole-Parmer GmbH, Germany) connected to a peristaltic pump (ISMATEC IPC series, Cole-Parmer GmbH, Germany). ISF extraction was performed just after the sensing procedure with the MIMN patch to minimize the possible alterations of the ISF either in quantity or quality.

IC measurements. For cations, the 850 Professional IC was equipped with a Metrosep C6 - 150/4.0 column set. 2.5 mmol/L HNO_3 was employed as eluent, and 0.7 mL/min was stated as flow rate. Carbonate buffer (1.0 mM sodium hydrogencarbonate / 3.2 mM sodium carbonate), and 100 mM H_2SO_4 were employed as eluent and suppressor solutions, respectively. 0.8 mL/min was stated as flow rate.

ISF samples were diluted in milli-Q water before IC analysis.

Serum samples were deproteinized by mixing four parts of pure ethanol solution with one part of serum. Serum ethanol mixtures were centrifuged at room temperature (24°C) and 6000 rpm for 10 min. Precipitation was assessed with a visual score.

Tables

Table S1. Compositions of the ion-selective membranes (ISMs) cocktails. Values are expressed in mg of the compound solved in 1 mL of THF. Values in brackets show the concentration of the corresponding compound in mmol kg^{-1} of membrane.

Membrane	Ionophore						Ion exchanger			Plasticizer		Polymer
	pH	K^+	Na^+	Ca^{2+}	Li^+	Cl^-	NaTFPB	KTClPB	TDMACl	DOS	NPOE	PU
HSM	0.99 (19.0)						0.76 (8.6)			65		33
KSM		2.20 (19.00)						0.50 (10.0)		65		33
NaSM			0.70 (7.0)				0.49 (5.5)			66		33
CaSM				1.00 (12.4)			0.86 (9.6)			66		33
LiSM					3.00 (80.1)		3.50 (40.9)			60		30
ClSM						1.83 (20.0)			0.29 (5.0)		66	33

Table S2. Interstitial fluid electrolyte values for human and rats.

Analyte	Reported Values, mM[a]	
	Rats[8, 9]	Humans[10, 11]
pH	7.2–7.5[12]	ca. 7.4[13]
Na^+	152.3	135.7
K^+	5.3	3.4
Ca^{2+}	1.6	1.1
Cl^-	109.0	–

[a]pH units for pH values

Table S3. Analytical parameters of the calibration of the MIMN patch for each ion analyte.

Analyte	RE	Sensitivity (mV dec^{-1})	Intercept (mV)	LOD (M)	LRR (M)	Response time (s)
pH	$RE_{comm.}$	–54.7 ± 0.7	586.4 ± 13.0	–	5.0 – 8.5	2.5 – 1.5
	RE-MN	–55.5 ± 0.3	697.2 ± 5.0	–	5.0 – 8.5	3.0 – 1.5
K^+	$RE_{comm.}$	52.1 ± 0.2	253.7 ± 9.0	1.1×10^{-5}	$3.2 \times 10^{-5} – 1.0 \times 10^{-1}$	5.0 – 3.0
	RE-MN	52.4 ± 0.2	414.2 ± 11.8	1.5×10^{-5}	$3.2 \times 10^{-5} – 1.0 \times 10^{-1}$	5.1 – 4.0
Na^+	$RE_{comm.}$	54.0 ± 0.6	308.2 ± 5.9	1.1×10^{-5}	$3.2 \times 10^{-5} – 3.2 \times 10^{-1}$	4.6 – 1.9
	RE-MN	54.4 ± 1.2	365.8 ± 14.1	1.8×10^{-5}	$3.2 \times 10^{-5} – 3.2 \times 10^{-1}$	5.0 – 4.0
Ca^{2+}	$RE_{comm.}$	26.4 ± 0.4	219.2 ± 2.8	3.8×10^{-6}	$3.2 \times 10^{-5} – 1.0 \times 10^{-1}$	4.8 – 2.8
	RE-MN	24.9 ± 0.5	137.5 ± 0.2	3.9×10^{-6}	$1.0 \times 10^{-5} – 1.0 \times 10^{-1}$	5.0 – 3.8
Li^+	$RE_{comm.}$	50.8 ± 0.4	339.6 ± 9.9	0.8×10^{-5}	$3.2 \times 10^{-5} – 1.0 \times 10^{-1}$	4.6 – 3.8
	RE-MN	51.8 ± 0.1	271.0 ± 5.5	1.2×10^{-5}	$3.2 \times 10^{-5} – 1.0 \times 10^{-1}$	5.0 – 4.2
Cl^-	$RE_{comm.}$	–55.5 ± 0.5	104.3 ± 13.8	8.1×10^{-6}	$3.2 \times 10^{-5} – 3.2 \times 10^{-1}$	4.8 – 2.4
	RE-MN	–47.2 ± 1.5	46.5 ± 0.9	8.4×10^{-6}	$1.0 \times 10^{-5} – 3.2 \times 10^{-1}$	5.1 – 3.2

Table S4. Comparison of the analytical performance of the MIMN with previously reported MNs systems.

Analyte	Sensing element	Slope (mV dec^{-1})	LOD (M)	LRR (M)	Ref
pH	Hydrogen ionophore I	54.6 ± 0.6	–	8.5 – 5.0	3
pH	PANI/MoS_2	56.6 ± 1.1	–	9 – 3	14
pH	ZnO	56.6 ± 1.1	–	9 – 2	15
pH	IrOx	52.4 ± 0.2	–	9 – 2	16
pH	Hydrogen ionophore I	55.5 ± 0.3	–	8.5 – 5.0	This work
K^+	Valinomycin	56.6 ± 0.2	$10^{-4.9}$	$10^{-4.2}$ – $10^{-1.1}$	17
K^+	Valinomycin	57.9	2.2 x 10^{-6}	10^{-5} – 10^{-2}	18
K^+	Valinomycin	50.03	–	0 – 1.5 x 10^{-3}	19
K^+	Valinomycin	52.4 ± 0.2	1.5 x 10^{-5}	3.2 x 10^{-5} – 1.0 x 10^{-1}	This work
Na^+	Sodium ionophore X	56.08	–	0 – 2 x 10^{-1}	19
Na^+	Sodium ionophore X	54.4 ± 1.2	1.8 x 10^{-5}	3.2 x 10^{-5} – 3.2 x 10^{-1}	This work
Ca^{2+}	Calcium ionophore IV	24.9 ± 0.5	3.9 x 10^{-6}	1.0 x 10^{-5} – 1.0 x 10^{-1}	This work
Li^+	Lithium ionophore VI	51.8 ± 0.1	1.2 x 10^{-5}	3.2 x 10^{-5} – 1.0 x 10^{-1}	This work
Cl^-	Chloride ionophore[2]	–47.2 ± 1.5	8.4 x 10^{-6}	1.0 x 10^{-5} – 3.2 x 10^{-1}	This work

Table S5. Between-electrodes reproducibility and repeatability evaluation of the MIMN patch.

Analyte	Sensitivity (% RSD)		Intercept (%RSD)	
	Repeatability	Reproducibility	Repeatability	Reproducibility
pH	0.5	3.6	0.7	3.6
K^+	0.4	0.6	2.8	2.6
Na^+	2.2	2.7	3.8	7.1
Ca^{2+}	2.1	3.4	2.9	5.6
Li^+	0.2	1.0	2.0	1.2
Cl^-	2.7	2.7	11.3	1.9

Table S6. Reversibility study with the MIMN patch. The corresponding pH and logarithm of the activity values are provided in brackets.

Analyte	RSD (%)			
pH	7.8 (8.0)	2.3 (7.0)	0.9 (6.0)	0.4 (5.0)
K^+	5.5 (–4.0)	2.4 (–3.0)	1.3 (–2.0)	0.2 (–1.1)
Na^+	5.7 (–4.0)	3.9 (–3.0)	1.5 (–2.0)	0.2 (–1.1)
Ca^{2+}	4.7 (–5.0)	3.7 (–4.0)	2.6 (–3.0)	0.6 (–2.1)
Li^+	0.8 (–5.0)	0.7 (–4.0)	0.6 (–3.0)	0.04 (–2.0)
Cl^-	31.3 (–4.0)	4.3 (–3.0)	2.7 (–2.0)	0.1 (–1.1)

Table S7. Selectivity coefficients for cations ($logK_{I,J}^{pot}$) calculated from the MIMN measurements together with those reported in the literature for analogous MNs and electrodes. I=main ion. J=interfering ion.

Ion (J)	$logK_{I,J}^{pot}$				
	**This paper**	**MN-ISE**	**Traditional ISE**		**Needed Value[c]**
	**pH MN**	**pH MN[3]**	**pH ISE[20]**	**Expected Concentration in ISF (mM)**	**pH**
K^+	–4.9 ± 0.07[a]	–6.7 ± 0.08	–9.3 ± 0.8	3.5	–1.8
Na^+	–4.7 ± 0.09[a]	–6.0 ± 0.09	–10.2 ± 0.1	135	–3.5
Ca^{2+}	–4.1 ± 0.06[a]	–5.0 ± 0.09	–10.4 ± 0.1	1.5	–2.9
Li^+	–5.1 ± 0.04[a]	–	–10.1 ± 0.1	1.5[b]	–2.0
	K^+ MN	K^+ MN[17]	K^+ ISE[20]		K^+
Na^+	–2.3 ± 0.02[a]	–3.2 ± 0.03	–2.8 ± 0.1	135	–1.7
Ca^{2+}	–2.8 ± 0.08[a]	–4.2 ± 0.04	–3.9 ± 0.1	1.54	0.3
Li^+	–3.2 ± 0.14[a]	–3.8 ± 0.03	–2.7 ± 0.1	1.5[b]	0.3
	Na^+ MN		Na^+ ISE[21]		Na^+
K^+	–1.7 ± 0.05		–2.7	3.5	1.9
Ca^{2+}	–6.4 ± 0.04		–6.6	1.5	2.5
Li^+	–2.5 ± 0.09		–	1.5[b]	2.0
	Ca^{2+} MN		Ca^{2+} ISE[22]		Ca^{2+}
K^+	–3.2 ± 0.06		–2.9 ± 0.1	3.5	–0.9
Na^+	–3.5 ± 0.19		–3.1 ± 0.1	135	–2.5
Li^+	–3.3 ± 0.4		–	1.5[b]	–0.5
	Li^+ MN		Li^+ ISE[23]		Li^+
K^+	–3.3 ± 0.10		–3.3	3.5	–0.3
Na^+	–2.3 ± 0.11		–3.1	135	–2.4
Ca^{2+}	–2.8 ± 0.08[b]		–2.9	1.5[b]	–0.1
	Cl^- MN		Cl^- ISE[24]		Cl^-
SO_4^{2-}	–[a]		0	0.5	2.4
$H_2PO_4^-$	–[a]		–1.2	2	2.3
HCO_3^-	–[a]		0	25	0.8

[a]These ions did not display a Nerstian slope and therefore, the calculated logarithmic selectivity coefficients are "biased" and can be only interpreted qualitatively as "apparent" values. [b]Therapeutic values were used as the expected concentration.[25] [c]Values calculated from the higher concentration of the interfering ion that can be found in ISF.

Table S8. Analytical parameters of the MINM patch observed from calibration in AISF (n=3) for each target analyte. The slope was calculated in the linear range of response. LOD=limit of detection. LRR=linear range of response.

Analyte	Slope (mV dec^{-1})	LOD (M)	LRR (M)
pH	–52.3 ± 0.8	–	8.5 – 5.0
K^+	54.3 ± 0.2	4.7 x 10^{-5}	1.0 x 10^{-4} – 1.0 x 10^{-1}
Na^+	56.0 ± 2.3	1.1 x 10^{-3}	3.2 x 10^{-3} – 3.2 x 10^{-1}
Ca^{2+}	25.5 ± 0.4	7.5 x 10^{-5}	3.2 x 10^{-4} – 1.0 x 10^{-1}
Li^+	50.1 ± 1.7	2.9 x 10^{-5}	1.0 x 10^{-4} – 1.0 x 10^{-1}
Cl^-	–25.8 ± 0.3	9.4 x 10^{-5}	3.2 x 10^{-4} – 3.2 x 10^{-1}

Table S9. Medium-term stability of the MINM patch in AISF.

Analyte	Concentration in the AISF	Drift (mV min^{-1})			Change in concentration in mM and %		
		15 min	30 min	120 min	15 min	30 min	120 min
pH [a]	7.5	–1.0	–0.5	–0.14	–0.02 (0.3 %)	0.04 (0.6 %)	–0.15 (2.5 %)
K^+	3.5	–0.02	–0.03	–0.06	3.4 (3%)	3.3 (7%)	2.6 (24%)
Na^+	140	–0.1	–0.1	–0.1	138 (1%)	136 (3%)	125 (11%)
Li^+	1.5	–0.2	–0.2	–0.6	1.4 (7%)	1.3 (13%)	0.9 (42%)
Ca^{2+}	1.5	–0.1	–0.2	–0.1	1.3 (16%)	0.9 (28%)	0.4 (74%)
Cl^-	146	0.01	0.02	0.04	140 (4%)	134 (8%)	104 (25%)

[a]pH is expressed in pH units.

Table S10. Comparison of the calibration parameters obtained before after three consecutive skin insertions.

Analyte	Sensibility (%RSD)	Intercept (%RSD)
pH	4.2	6.0
K^+	3.0	3.1
Na^+	3.1	9.4
Ca^{2+}	5.0	10.2
Li^+	5.1	4.4
Cl^-	6.2	9.1

Table S11. *Ex vivo* experiments (transdermal detection with the MIMN patch and analysis of collected dermal fluid with IC): pH and ions concentrations in pieces of rat skin conditioned overnight in different solutions. Values in brackets correspond to the ion concentration in the external solution after the overnight conditioning.

Target Ion	Skin number (#)	Conditioning solution, mM	Intradermal concentration, mM		
			MIMN	Reference method[a]	% Diff[c]
pH	#1	7.6 (7.5)[b]	7.4 ± 0.2[b]	7.45[b]	1
	#2	6.5 (6.51)[b]	6.4 ± 0.1[b]	6.51[b]	2
K^+	#3	0.1 (2.2)	1.1 ± 0.1	1.3	14
	#4	0.1 (2.2)	5.4 ± 0.1	5.1	6
	#5	10.0 (11.1)	12.3 ± 2.0	12.0	2
Na^+	#6	0.1 (2.8)	1.3 ± 0.1	1.1	18
	#7	0.1 (2.8)	3.1 ± 1.0	2.7	15
	#8	10.0 (15.8)	14.5 ± 1.1	12.3	18
Ca^{2+}	#9	0.1 (0.08)	0.08 ± 0.01	0.09	11
	#10	10.0 (9.4)	4.0 ± 0.5	3.7	9
Li^+	#11	0.1 (0.08)	0.07 ± 0.01	0.09	20
	#12	0.1 (0.08)	0.06 ± 0.01	0.07	14
	#13	1.0 (0.9)	0.80 ± 0.02	0.9	11
	#14	1.0 (0.9)	0.40 ± 0.03	0.5	20
	#15	10.0 (9.5)	3.1 ± 0.7	3.5	11
	#16	10.0 (9.5)	9.9 ± 0.9	11.1	11
Cl^-	#17	10.0 (8.9)	11.1	10.7	4

[a]Micro pH electrode for pH and IC for the rest of ions.

[b]pH units.

[c]% of difference between the result observed with the MIMN and that with the IC.

Table S12. Characteristics of the rats employed in *in vivo* assays and the corresponding samples collection.

Rat#	Age (months)	Gender	MN that functioned	Subcutaneous pH?	Blood	Serum
1	9	F	pH, K^+	–	–	–
2	9	M	pH, Cl^-	–	–	Yes (anions)
3	9	F	pH, Cl^-	Yes	–	Yes (anions & cations)
4	4	M	pH, Na^+, K^+, Ca^{2+}, Cl^-	Yes	Yes	Yes (anions & cations)
5	4	M	pH, Na^+, K^+, Ca^{2+}, Cl^-	Yes	Yes	Yes (anions & cations)
6	4	M	pH, Na^+, K^+, Ca^{2+}, Cl^-	–[a]	Yes	Yes (anions & cations)

[a]The pH could not be measured because the subcutaneous part of the back was dry when the measurement was attempted.

Figures

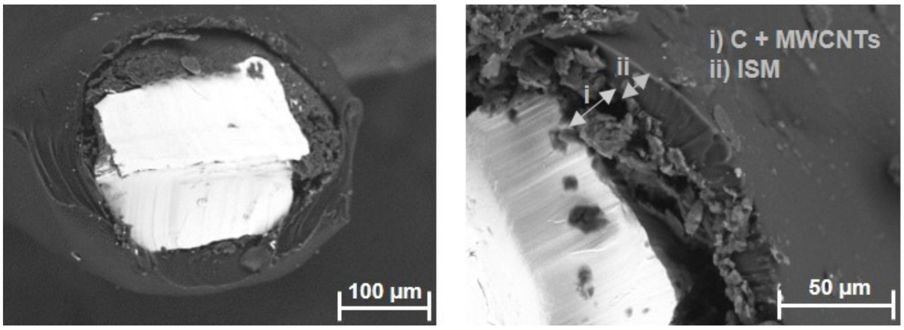

Figure S1. SEM images of a cross-section of the WE for pH (left). Magnification of the cross-section (right).

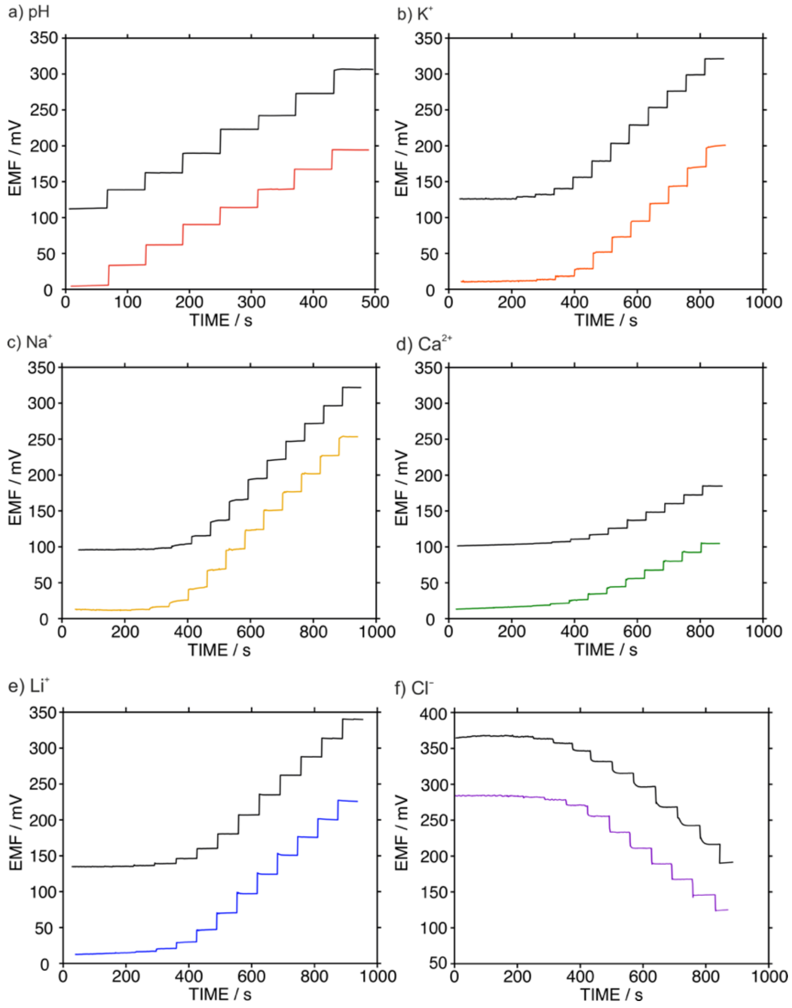

Figure S2. Dynamic potentiometric response for increasing ions activity of the microneedle working electrodes against two different reference electrodes: Ag/AgCl commercial reference electrode (color) and microneedle-based reference electrode (black) for increasing ions activity. (a) pH; (b) K^+, (c) Na^+, (d) Ca^{2+}, (e) Li^+ and (f) Cl^-.

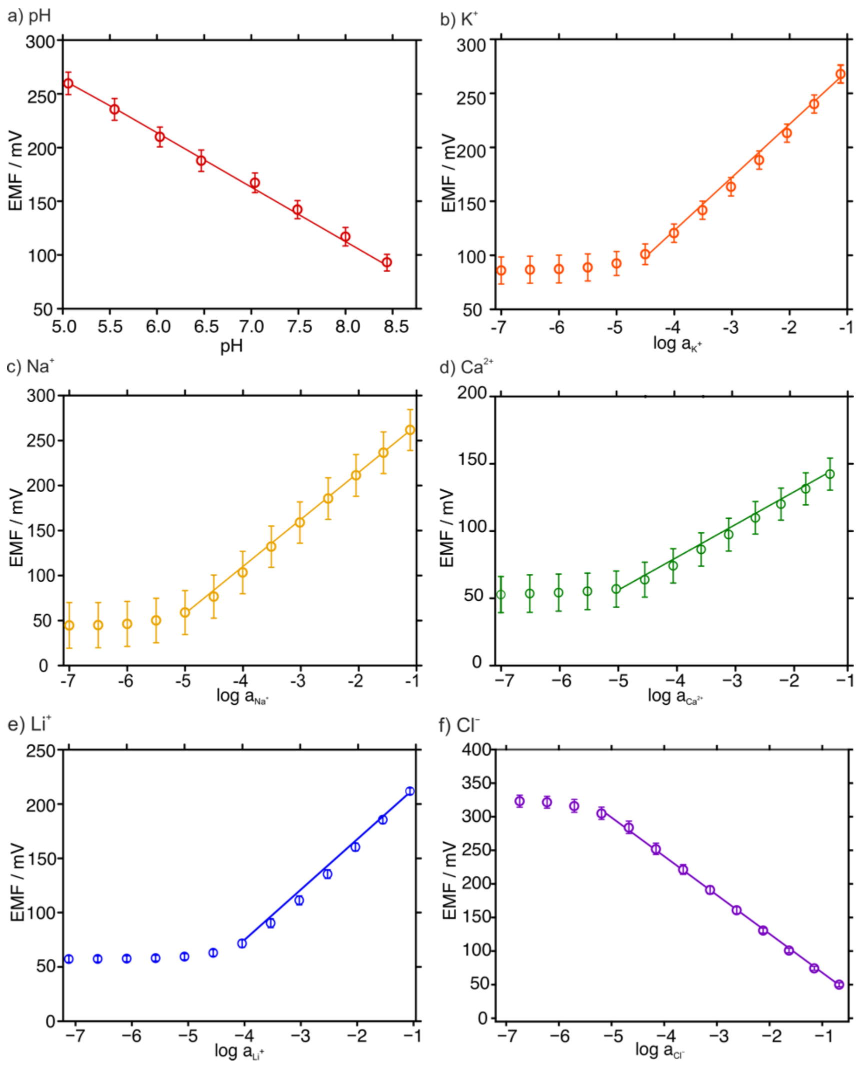

Figure S3. Average calibration graph obtained with three similar MIMN in water. (a) pH; (b) K^+, (c) Na^+, (d) Ca^{2+}, (e) Li^+ and (f) Cl^-.

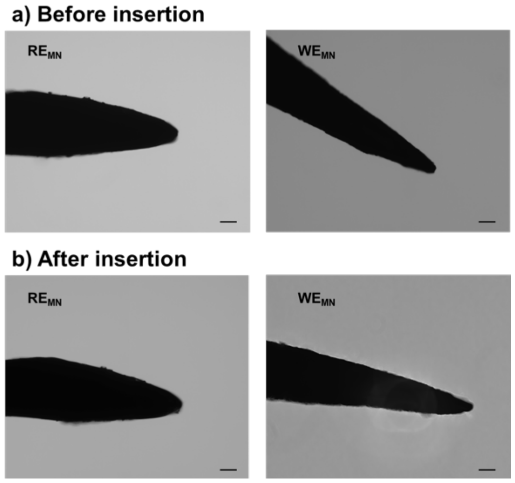

Figure S4. Optical microscopy images of MIMN sensors (RE-MN, and WE-MN) components before (a) and after (b) 3 insertions into rat skin. Scale bar: 100 μm.

References

1. Yuan, D.; Anthis, A. H.; Ghahraman Afshar, M.; Pankratova, N.; Cuartero, M.; Crespo, G. A.; Bakker, E., All-solid-state potentiometric sensors with a multiwalled carbon nanotube inner transducing layer for anion detection in environmental samples. *Anal Chem* **2015,** *87* (17), 8640-5.
2. Busschaert, N.; Wenzel, M.; Light, M. E.; Iglesias-Hernández, P.; Pérez-Tomás, R.; Gale, P. A., Structure–Activity Relationships in Tripodal Transmembrane Anion Transporters: The Effect of Fluorination. *J Am Chem Soc* **2011,** *133* (35), 14136-14148.
3. Garcia-Guzman, J. J.; Perez-Rafols, C.; Cuartero, M.; Crespo, G. A., Toward In Vivo Transdermal pH Sensing with a Validated Microneedle Membrane Electrode. *ACS Sens* **2021,** *6* (3), 1129-1137.
4. Buck, R. P.; Lindner, E., Recommendations for nomenclature of ionselective electrodes (IUPAC Recommendations 1994). *Pure and Applied Chemistry* **1994,** *66* (12), 2527-2536.
5. Lindner, E.; Umezawa, Y., Performance evaluation criteria for preparation and measurement of macro- and microfabricated ion-selective electrodes (IUPAC Technical Report). *Pure and Applied Chemistry - PURE APPL CHEM* **2008,** *80*, 85-104.
6. Guinovart, T.; Crespo, G. A.; Rius, F. X.; Andrade, F. J., A reference electrode based on polyvinyl butyral (PVB) polymer for decentralized chemical measurements. *Anal Chim Acta* **2014,** *821*, 72-80.
7. Meier, P. C., Two-parameter debye-hückel approximation for the evaluation of mean activity coefficients of 109 electrolytes. *Analytica Chimica Acta* **1982,** *136*, 363-368.
8. Bretag, A. H., Synthetic interstitial fluid for isolated mammalian tissue. *Life Sci* **1969,** *8* (5), 319-29.
9. Gilányi, M.; Ikrényi, C.; Fekete, J.; Ikrényi, K.; Kovách, A. G., Ion concentrations in subcutaneous interstitial fluid: measured versus expected values. *Am J Physiol* **1988,** *255* (3 Pt 2), F513-9.
10. Fogh-Andersen, N.; Altura, B. M.; Altufu, B. T.; Siggaard-Andersen, O., Changes in plasma ionized calcium and magnesium in blood donors after donation of 450 mL blood. Effects of hernodilution and Donnan equilibrium. *Scandinavian Journal of Clinical and Laboratory Investigation* **1996,** *56* (sup224), 245-250.
11. Fogh-Andersen Niels, N. M. A., B.T. Altura, O. Siggard-Anderssen, Composition of interstitial fluid, Gen. *Clinical chemistry.* **1995,** *41*, 1522.
12. Marunaka, Y., Roles of interstitial fluid pH in diabetes mellitus: Glycolysis and mitochondrial function. *World J Diabetes* **2015,** *6* (1), 125-35.
13. Marunaka, Y., Roles of interstitial fluid pH and weak organic acids in development and amelioration of insulin resistance. *Biochemical Society Transactions* **2021,** *49* (2), 715-726.
14. Zhou, J. X.; Ding, F.; Tang, L. N.; Li, T.; Li, Y. H.; Zhang, Y. J.; Gong, H. Y.; Li, Y. T.; Zhang, G. J., Monitoring of pH changes in a live rat brain with MoS2/PAN functionalized microneedles. *Analyst* **2018,** *143* (18), 4469-4475.
15. Mani, G. K.; Miyakoda, K.; Saito, A.; Yasoda, Y.; Kajiwara, K.; Kimura, M.; Tsuchiya, K., Microneedle pH Sensor: Direct, Label-Free, Real-Time Detection of Cerebrospinal Fluid and Bladder pH. *ACS Appl Mater Interfaces* **2017,** *9* (26), 21651-21659.
16. Zuliani, C.; Ng, F. S.; Alenda, A.; Eftekhar, A.; Peters, N. S.; Toumazou, C., An array of individually addressable micro-needles for mapping pH distributions. *Analyst* **2016,** *141* (15), 4659-4666.
17. Parrilla, M.; Cuartero, M.; Padrell Sanchez, S.; Rajabi, M.; Roxhed, N.; Niklaus, F.; Crespo, G. A., Wearable All-Solid-State Potentiometric Microneedle Patch for Intradermal Potassium Detection. *Anal Chem* **2019,** *91* (2), 1578-1586.

18. Miller, P. R.; Xiao, X.; Brener, I.; Burckel, D. B.; Narayan, R.; Polsky, R., Microneedle-based transdermal sensor for on-chip potentiometric determination of K(+). *Adv Healthc Mater* **2014,** *3* (6), 876-81.
19. Li, H.; Wu, G.; Weng, Z.; Sun, H.; Nistala, R.; Zhang, Y., Microneedle-Based Potentiometric Sensing System for Continuous Monitoring of Multiple Electrolytes in Skin Interstitial Fluids. *ACS Sens* **2021,** *6* (6), 2181-2190.
20. Novell, M.; Parrilla, M.; Crespo, G. A.; Rius, F. X.; Andrade, F. J., Paper-based ion-selective potentiometric sensors. *Anal Chem* **2012,** *84* (11), 4695-702.
21. Lim, H. R.; Lee, S. M.; Mahmood, M.; Kwon, S.; Kim, Y. S.; Lee, Y.; Yeo, W. H., Development of Flexible Ion-Selective Electrodes for Saliva Sodium Detection. *Sensors (Basel)* **2021,** *21* (5).
22. Lindfors , T.; Sundfors, F.; Höfler , L.; Gyurcsányi , R. E., The Water Uptake of Plasticized Poly(vinyl chloride) Solid-Contact Calcium-Selective Electrodes. *Electroanalysis* **2011,** *23* (9), 2156-2163.
23. Novell, M.; Guinovart, T.; Blondeau, P.; Rius, F. X.; Andrade, F. J., A paper-based potentiometric cell for decentralized monitoring of Li levels in whole blood. *Lab Chip* **2014,** *14* (7), 1308-14.
24. Pankratova, N.; Cuartero, M.; Jowett, L. A.; Howe, E. N. W.; Gale, P. A.; Bakker, E.; Crespo, G. A., Fluorinated tripodal receptors for potentiometric chloride detection in biological fluids. *Biosens Bioelectron* **2018,** *99*, 70-76.
25. Nolen, W. A.; Licht, R. W.; Young, A. H.; Malhi, G. S.; Tohen, M.; Vieta, E.; Kupka, R. W.; Zarate, C.; Nielsen, R. E.; Baldessarini, R. J.; Severus, E., What is the optimal serum level for lithium in the maintenance treatment of bipolar disorder? A systematic review and recommendations from the ISBD/IGSLI Task Force on treatment with lithium. *Bipolar Disord* **2019,** *21* (5), 394-409.
